# Influence of *Schistosoma mansoni* and Hookworm Infection Intensities on Anaemia in Ugandan Villages

**DOI:** 10.1371/journal.pntd.0004193

**Published:** 2015-10-29

**Authors:** Goylette F. Chami, Alan Fenwick, Erwin Bulte, Andreas A. Kontoleon, Narcis B. Kabatereine, Edridah M. Tukahebwa, David W. Dunne

**Affiliations:** 1 Department of Land Economy, University of Cabridge, Cambridge, United Kingdom; 2 Department of Pathology, University of Cambridge, Cambridge, United Kingdom; 3 Schistosomiasis Control Initiative, Imperial College London, London, United Kingdom; 4 Development Economics Group, Wageningen University, Wageningen, Netherlands; 5 Uganda Ministry of Health, Vector Control Division, Bilharzia and Worm Control Programme, Kampala, Uganda; George Washington University, UNITED STATES

## Abstract

**Background:**

The association of anaemia with intestinal schistosomiasis and hookworm infections are poorly explored in populations that are not limited to children or pregnant women.

**Methods:**

We sampled 1,832 individuals aged 5–90 years from 30 communities in Mayuge District, Uganda. Demographic, village, and parasitological data were collected. Infection risk factors were compared in ordinal logistic regressions. Anaemia and infection intensities were analyzed in multilevel models, and population attributable fractions were estimated.

**Findings:**

Household and village-level predictors of *Schistosoma mansoni* and hookworm were opposite in direction or significant for single infections. *S*. *mansoni* was found primarily in children, whereas hookworm was prevalent amongst the elderly. Anaemia was more prevalent in individuals with *S*. *mansoni* and increased by 2.86 fold (p-value<0.001) with heavy *S*. *mansoni* infection intensity. Individuals with heavy hookworm were 1.65 times (p-value = 0.008) more likely to have anaemia than uninfected participants. Amongst individuals with heavy *S*. *mansoni* infection intensity, 32.0% (p-value<0.001) of anaemia could be attributed to *S*. *mansoni*. For people with heavy hookworm infections, 23.7% (p-value = 0.002) of anaemia could be attributed to hookworm. A greater fraction of anaemia (24.9%, p-value = 0.002) was attributable to heavy hookworm infections in adults (excluding pregnant women) as opposed to heavy hookworm infections in school-aged children and pregnant women (20.2%, p-value = 0.001).

**Conclusion:**

Community-based surveys captured anaemia in children and adults affected by *S*. *mansoni* and hookworm infections. For areas endemic with schistosomiasis or hookworm infections, WHO guidelines should include adults for treatment in helminth control programmes.

## Author Summary

Anaemia remains an intractable public health problem in sub-Saharan Africa (SSA), owing largely to its complex and multifactorial causes. The absolute number of anaemia cases has increased by 50% in SSA from the year 1990 to 2010. To administer the appropriate treatment, identification of anaemia attributable to specific causes is required. In this paper, we assessed the relative contribution to anaemia of intestinal schistosomiasis (*Schistosoma mansoni*) and hookworm (a soil-transmitted helminth). The effect of *S*. *mansoni* on anaemia is controversial; this infection has not been found to consistently affect anaemia. However, hookworm has widely been shown to increase anaemia risk. Yet, limited information is available about the effect of *S*. *mansoni* and hookworm infections in populations that are not limited to children or pregnant women. Without this information, current World Health Organization guidelines for the treatment of anaemia due to intestinal helminths neglect a large portion of the general population by age and gender. To address this lack of information, we conducted a communitywide assessment of the effects of *S*. *mansoni* and hookworm infections on anaemia in rural Uganda.

## Introduction

Anaemia remains an intractable public health problem in sub-Saharan Africa (SSA). Children younger than five years and pregnant women are the focus of most epidemiological surveys, as prevalence is highest amongst these groups [1–11]. In SSA, over 64% of children younger than five years and 55% of pregnant women are estimated to have anaemia [2]. Designing treatment packages for anaemia is complex, owing largely to its multifactorial aetiology. These causes include genetic haemoglobinopathies, haemorrhage, bacteraemia, deficiencies of iron or other micronutrients including copper, folate, vitamins A and B_12_, and parasitic infections of malaria, schistosomiasis, and hookworm [12, 13]. If left untreated, anaemia can reduce work capacity, decrease immunogenicity, and impair cognitive or motor development [3–6, 14].

In this paper, we focus on the relationship between anaemia and intestinal helminths. The relevance of intestinal schistosomiasis (*S*. *mansoni*) for anaemia remains controversial, including its causal mechanism [9, 10]. Recent studies in SSA [15–17] focused on preschool children or adults in a single village and found no correlation between *S*. *mansoni* infection and anaemia. Yet, in school-aged children and pregnant women, heavy *S*. *mansoni* infection intensity has been shown to increase anaemia risk [18, 19]. The relationship of hookworm infection and intestinal blood loss is well established [20]. This association is intensity-dependent with moderate to heavy infections required for anaemia [21–23]. In SSA, anaemia attributable to hookworm has been shown to be 18% in preschool children [21], 5%-25% in school-aged individuals [22, 23], and 28% in pregnant women [24].

The associations of anaemia with *S*. *mansoni* and hookworm infections have been studied in the same population under two contexts: efficacy of anthelminthic treatment and polyparasitism. Amongst infected schoolchildren, separate treatments with praziquantel for *S*. *mansoni* and albendazole or mebendazole for hookworm have been found to reduce anaemia [19, 25, 26]. Although individually relevant in SSA, moderate to heavy co-intensities of *S*. *mansoni* and hookworm have been found to have no significant effect on anaemia in individuals under six years in Uganda [16] and school-aged children in Rwanda [27].

With previous epidemiological surveys in SSA focused on young children and pregnant women, current World Health Organization (WHO) guidelines for treating anaemia due to hookworm neglect community-based samples [11, 28]. Additional community-based research is required to assess if guidelines should be established for treating anaemia attributable to *S*. *mansoni* infection [11, 28]. There is a need to identify the levels of *S*. *mansoni* and hookworm infection intensities associated with anaemia in populations, which are not limited to young children, school-based samples, or pregnant women. Moreover, it is unknown whether *S*. *mansoni* and hookworm infections influence anaemia prevalence in the same age group for community-based samples. To prioritize treatment strategies for anaemia, there also is a need to accurately quantify the proportion of anaemia attributable to *S*. *mansoni* and hookworm infections in community-based samples. Herein, we present a large-scale, community-based investigation of anaemia and intestinal helminths in SSA.

## Methods

### Study site and village sampling

Data were collected from August-September 2013 in 30 villages of Mayuge District, Uganda. Annual and en masse treatments with praziquantel for *S*. *mansoni* and albendazole for hookworm began in the study area in 2003 and were scaled-up to all sub-counties in the study district in 2004 by the national helminth control programme [29, 30]. An area within five kilometers of Lake Victoria in Mayuge District was chosen, as previous nationwide surveys [31, 32] showed both *S*. *mansoni* and hookworm infection prevalence exceeds 50% for schoolchildren aged 5–21 years. In these surveys, the prevalence of coinfection was unknown. Accordingly, this area, which is within five kilometres of Lake Victoria in Mayuge District, was classified as a high-risk area for schistosomiasis infections. Every person aged five years and older was treated annually with praziquantel. If the area was not high-risk, praziquantel would only be available for children through primary schools. Adults are generally not treated through mass drug administration (MDA) with albendazole for hookworm infections [33]. However, our study area was endemic for lymphatic filariasis and all individuals aged five years and older were treated annually with a package of albendazole and ivermectin. MDA is the only substantial strategy for controlling these helminth infections, thus this context must be studied to reflect the true public health situation. The Ugandan national control programme aims to administer annual treatment for schistosomiasis and twice yearly treatment for soil-transmitted helminths. However, due to in-country delays, Mayuge District did not receive MDA for 1.5 years prior to our study.

Thirty villages were selected for this study as follows. In February 2013, 41 villages were visited in this catchment. For each of the eight sub-counties of Mayuge District, the district health officer identified one village with known *S*. *mansoni* transmission. At least four villages in closest physical proximity to these initial eight villages were sampled. One cluster of 3–8 villages was sampled in each sub-county. In addition to the collection of village waypoints, researchers drew maps with village chairmen to identify locations of taps, rice paddies, standing seasonal water, public latrines, homes, and primary schools. These maps were used to select six village clusters from the initial eight sub-counties that were similar in size, language, infrastructural development, quality of homes, and distance to established towns.

### Participant sampling

The sample included 1,832 individuals aged 5–90 years (Mean 24.31, Std. Dev. 16.90, includes one four year-old) from 916 households. Village health team members selected at least 30 households and two people per household in their village. To ensure men participated, health team members were instructed to stratify participants by gender and familial position, e.g. mothers with daughters, mothers with sons, fathers with daughters, and fathers with sons. This stratification enabled a multilevel analysis (described below) to account for latent, unobserved household or village effects on anaemia. Child participants were required to be at least five years, which is the minimum age to treat *S*. *mansoni* infections with praziquantel [33]. Moreover, sampling of children focused on ages 10–14, which is where the peak intensity of *S*. *mansoni* infection occurs in most endemic areas [34]. There were no other restrictions on the child, who was chosen by the village health team member.

Lastly, at-risk groups of fishermen (*S*. *mansoni*) and farmers (hookworm) were sampled. Stratification by occupation was conducted to ensure representation of occupations that were characteristic of the high prevalence villages. This approach was needed, as fishermen and farmers leave the village during the day to work and may otherwise be underrepresented. This sampling did not affect the internal validity of the data and should not have an effect on the generalizability of the study if endemic, high-risk villages are of interest. However, given the number of villages, a complete demographic census was not performed and should be considered in future work. A random non-stratified sampling strategy may be preferred for areas with low *S*. *mansoni* or hookworm infection prevalence.

### Parasitology, anaemia, and household data

Each participant provided one stool sample. Stool samples were immediately processed upon receipt and standard Kato-Katz methodology for two thick smear slides (41.7 mg) [35] was used. Technicians read slides within 30 minutes of preparation for the presence of hookworm eggs and slides were reread 24 hours after preparation to count eggs of *S*. *mansoni* and other STHs. Ten percent of slides were retained for *S*. *mansoni* quality control egg counts by a senior technician, who confirmed the accuracy of the field readings. Egg counts were multiplied by 24 then averaged to represent *S*. *mansoni* and hookworm infection intensities as the number of eggs per gram (EPG). To assess anaemia, every participant provided a fingerprick blood sample for the measurement of haemoglobin (Hb) (g/L) in Hemocue Haemoglobinometers (Radiometer Group, Sweden). A short questionnaire was administered to all adults, who also answered on behalf of the child. Participants provided demographic information, the last incident of malaria, and income-earning occupation (if any) of the household head.

### Statistical analysis

The data were analyzed in Stata v.12.1. The main variables used to examine anaemia were as follows. WHO categories for light (1–99 EPG), moderate (100–399 EPG), and heavy (400+ EPG) *S*. *mansoni* infection intensity were used [36]. These values also were applied to classify hookworm, as 400 or more EPG represented the top 10^th^ percentile of hookworm infection intensity measured in our study. This method of hookworm infection classification also is employed in Pullan et al. [22]. Anaemic individuals were classified according to WHO guidelines for Hb by age and gender [11]. All models in this paper excluded basic age-gender-infection or *S*. *mansoni*-hookworm interactions, as there was insufficient support of model improvement from likelihood ratio tests (p-value>0.05). Descriptive statistics of variables used in analyses are provided in Tables A-D in S1 File.

To understand the distribution of infection intensities and to check the robustness of this data with known epidemiological studies, the risk factors of *S*. *mansoni* and hookworm infections were compared in ordinal logistic regressions [37]. Infection categories were ranked from uninfected to heavy infections and used as dependent variables. In these models, the relevance of age, gender, household head occupation, and village-level factors were examined for infection intensity. Village predictors included a continuous variable for the number of houses and the following binary indicators for environmental factors within the village: beach on Lake Victoria, lake site only (no beach, only small boat landing site to Lake Victoria), a rice paddy (rice farm), and three or more village roads. Additionally, a binary indicator was used that was equal to one if the distance of the village centre was greater than 0.50 kilometres to Lake Victoria. Standard errors were adjusted with robust sandwich estimators at the household level [38]. Ordinal logistic regressions present cumulative probabilities and assume proportional odds of being in higher outcome categories. There was sufficient evidence that age, the occupations of business and rice farmer, and the distance of the village centre to Lake Victoria violated this assumption in the hookworm model [39]. Using methods described in Peterson and Harrell [40] and Williams [39], these variables were allowed to vary across hookworm infection levels and the associated beta estimates were provided.

Anaemia status was analyzed with a multilevel logistic regression that controlled for household and village variance. General linear and latent mixed models were used [41]. Infection categories were incorporated as predictors of anaemia and presented as binary variables of low, moderate, and heavy *S*. *mansoni* or hookworm infections (with uninfected as the base category). Gender, dummies for household head occupation, and age (in years) were included as covariates. Other covariates of age grand-mean-centered squared, self-reported malaria (individual received antimalarial medicines from government or private health clinic within the past six months), and village-level factors were tested by forward selection. Univariate and empty logistic models were compared. If the likelihood ratio test was significant (p-value<0.05), the variable was used (Table E in S1 File). A crude global R^2^ was calculated using the square of the correlation between fitted and actual anaemia values [42]. Although this method is used in linear regression, the crude global R^2^ does not provide insight into the proportional reduction in error variance explained by the full model. Accordingly, more robust calculations of R^2^ were calculated using recent methods of Nakagawa and Schielzeth [43]. For variation explained by only the fixed component and full model, the marginal R^2^ and conditional R^2^, respectively, were estimated. Intra-class correlation (ICC) coefficients were calculated as described in McGraw and Wong [44].

The assessment of coinfection and anaemia only differed from the aforementioned anaemia status model with respect to the infection variables used; otherwise, all other variables and model specifications were the same. *S*. *mansoni* and hookworm coinfection categories were presented as binary indicators and used as predictors of anemia. The base category for each coinfection indicator included no infection (zero EPG) and single infections of *S*. *mansoni* or hookworm. Four categories were used to classify coinfection intensity. Light, moderate, and heavy coinfections were defined, respectively, as at least 1–99 EPG, 100–399 EPG, and 400+ EPG of both *S*. *mansoni* and hookworm infections.

To quantify the impact of *S*. *mansoni* and hookworm infection on anaemia, population attributable fractions (PAF) were estimated using maximum likelihood procedures described in Greenland and Drescher [45, 46]. This method adjusts for covariates and calculates PAF confidence intervals (CI), but random effects or latent variable distributions in the data cannot be preserved. Accordingly, logistic regressions were used with the same variables as the multilevel anaemia model and clustered standard errors at the household level [38]. Infection intensities found significant in the anaemia logistic model were examined. Two PAF scenarios were estimated: elimination and intensity reduction. These scenarios were assessed across the whole study sample, a sub-population of heavily infected individuals, and a sub-group that excluded school-aged children and pregnant women.

### Ethics

This study was reviewed and approved by the Uganda National Council of Science and Technology (SS3082), Office of the President in Uganda (SS3082), and Cambridge University Human Biological Research Ethics Committee (HBREC2013.10). Written informed consent was obtained from participants or their guardians. For adults or guardians who indicated they were unable to write or who preferred to provide fingerprints, verbal informed consent and a fingerprint signature were obtained (reviewed and approved in SS3082 and HBREC2013.10).

## Results

### Distribution of anaemia, *S*. *mansoni*, and hookworm by age

**Fig 1:**
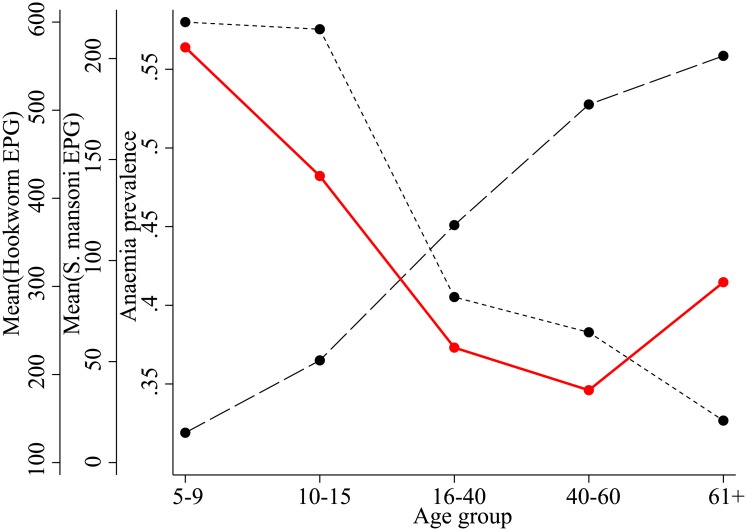
Overlaid plot of *S*. *mansoni* and hookworm infection intensities and anaemia prevalence against age. The first axis and red line (solid line) represent anaemia prevalence for each age group. The second axis and short dashed line portray the arithmetic mean of *S*. *mansoni* eggs per gram (EPG) for each age group. Similarly, the third axis and long-dashed line represent the arithmetic mean of hookworm EPG by age group.

Prevalence (at least one detectable EPG) of *S*. *mansoni* and hookworm was 36.4% (667/1832) and 40.5% (741/1832), respectively, which included 13.1% (240/1832) of the population with coinfection. Overall anaemia prevalence was 44.4% (813/1832). Anaemia prevalence coincided with the highest average *S*. *mansoni* or hookworm EPG (Tables C and D in S1 File). As shown (Fig 1), anaemia prevalence formed a U-curve, being highest in children and the elderly. These age groups harboured the heaviest *S*. *mansoni* and hookworm infections, respectively. Anaemia prevalence was at least 34.6% in all age groups. Although coinfections of *S*. *mansoni* and hookworm were observed in 13.1% of the population, only 0.55% (10/1832) of the population had coinfections with heavy infection intensity of both *S*. *mansoni* and hookworm (Table 1).

**Table 1:** The number of individuals by *S*. *mansoni* and hookworm infection intensities.

		Hookworm infection intensity				Total
		Uninfected^a^	Low^b^	Moderate^c^	Heavy^d^	
***S*. *mansoni* infection intensity**	**Uninfected** ^**a**^	664	204	150	147	1165
		(36.24%)	(11.14%)	(8.19%)	(8.02%)	(63.59%)
	**Low** ^**b**^	222	72	41	41	376
		(12.12%)	(3.93%)	(2.24%)	(2.24%)	(20.52%)
	**Moderate** ^**c**^	107	20	13	12	152
		(5.84%)	(1.09%)	(0.71%)	(0.66%)	(8.3%)
	**Heavy** ^**d**^	98	21	10	10	139
		(5.35%)	(1.15%)	(0.55%)	(0.55%)	(7.59%)
	**Total**	1091	317	214	210	1832
		(59.55%)	(17.30%)	(11.68%)	(11.46%)	(100%)

^a^ 0 eggs per gram (EPG)

^b^ 1–99 EPG

^c^ 100–399 EPG

^d^ 400+ EPG

### Transmission determinants of *S*. *mansoni* and hookworm infections

At the village level, mean *S*. *mansoni* EPG and mean hookworm EPG were inversely related (Spearman rho -0.407, p-value<0.0001). Similarly, most communities did not have high prevalence of both infections (Spearman rho -0.562, p-value<0.0001). In ordinal logistic models in Table 2, *S*. *mansoni* and hookworm showed no overlap of infection determinants.

**Table 2:** Determinants of *S*. *mansoni* and hookworm infection.

Variable	A) Dependent variable of S. mansoni intensity level^a^					B) Dependent variable of hookworm intensity level^a^				
	Odds ratio	Clustered Std. Err.	p-value	95% CI		Odds ratio	Clustered Std. Err.	p-value	95% CI	
Age	0.979	0.003	<0.001	0.972	0.985	1.012	0.003	<0.001	1.006	1.018
Female	0.685	0.074	<0.001	0.554	0.847	1.427	0.144	<0.001	1.171	1.739
Business (owns small shop)	1.388	0.289	0.115	0.923	2.087	0.346	0.087	<0.001	0.211	0.565
Fishing (fishermen and fishmongers)	2.700	0.542	<0.001	1.822	4.001	0.757	0.159	0.185	0.501	1.143
Rice farmer	0.905	0.140	0.517	0.668	1.225	1.130	0.164	0.398	0.851	1.502
School teacher	0.607	0.268	0.257	0.255	1.440	0.745	0.454	0.629	0.226	2.459
Other employment	1.083	0.227	0.702	0.719	1.632	0.905	0.179	0.613	0.613	1.334
Rice paddy (large rice farm) within village	0.708	0.134	0.069	0.488	1.027	1.302	0.203	0.091	0.959	1.768
Beach on Lake Victoria within village	2.059	0.311	<0.001	1.532	2.767	0.755	0.112	0.059	0.564	1.010
Lake site only within village (no beach, but small boat landing site on Lake Victoria)	1.126	0.153	0.384	0.862	1.469	1.076	0.119	0.505	0.867	1.336
Village centre greater than 0.50 km to Lake Victoria	0.321	0.041	<0.001	0.250	0.412	1.489	0.181	0.001	1.174	1.889
3 or more roads within village	0.798	0.116	0.121	0.599	1.062	1.937	0.239	<0.001	1.521	2.466
Natural log of total houses in village	1.572	0.203	<0.001	1.221	2.024	1.064	0.122	0.590	0.850	1.332
**Ancillary parameters (Model cuts)**						**Constants (Model cuts)**				
Low intensity^b^	1.550	0.774		0.033	3.067	0.152	0.103	0.005	0.040	0.574
Moderate intensity^c^	2.875	0.779		1.349	4.401	0.044	0.030	<0.001	0.012	0.168
Heavy intensity^d^	3.817	0.781		2.287	5.347	0.011	0.008	<0.001	0.003	0.045
					**Moderate hookworm intensity** ^**e**^					
					Age	1.020	0.003	<0.001	1.013	1.026
					Business	0.547	0.153	0.031	0.315	0.947
					Rice farmer	0.808	0.132	0.191	0.587	1.112
					Village centre more than 0.50 km to Lake Victoria	1.953	0.282	<0.001	1.472	2.591
					**Heavy hookworm intensity** ^**e**^					
					Age	1.026	0.004	<0.001	1.018	1.034
					Business	0.786	0.268	0.481	0.403	1.534
					Rice farmer	0.901	0.185	0.612	0.603	1.347
					Village centre more than 0.50 km to Lake Victoria	2.599	0.532	<0.001	1.741	3.881
**Obs.** 1832						**Obs.** 1832				

^**a**^ Multivariate ordinal logistic regression with standard errors adjusted for 916 household clusters.

^**b**^1-99 eggs per gram (EPG)

^**c**^ 100–399 EPG

^**d**^400+ EPG

^**e**^The variables that do not satisfy the proportional odds assumption in the hookworm model were allowed to vary for each outcome. In Panel B of the main table, the coefficients of these variables represent the odds of moving from no hookworm infection to any hookworm infection (at least one EPG). The additional coefficients below the main table represent the odds of moving from low hookworm infection to moderate or heavy hookworm infection and the odds of changing from moderate hookworm infection to heavy hookworm infection.

Occupations are represented at the household level and indicate the employment of the household head.

Any variables that were significant in both models were opposite in direction. A one-year increase in age or being female decreased the likelihood of *S*. *mansoni* infection by 1.98% (p-value<0.001) and 31.5% (p-value<0.001), but increased the odds of hookworm infection by 1.2% (p-value<0.001) and 42.7% (p-value<0.001), respectively. Belonging to a community where the village centre is farther than 0.50 kilometres from Lake Victoria decreased the likelihood of *S*. *mansoni* infection by 67.9% (p-value<0.001) and increased the probability of hookworm infection by 48.9% (p-value = 0.001).

For *S*. *mansoni*, variables measuring water contact increased the probability of infection. Fishing occupations increased the likelihood of *S*. *mansoni* 2.70 fold (p-value<0.001) when compared to households with an unemployed person or subsistence farmer as the household head. Individuals living in a village with a beach were 2.06 times (p-value<0.001) more likely to have *S*. *mansoni* infections than individuals in a village without a freshwater site. Additionally, having more homes in the village increased the odds of heavy *S*. *mansoni* infection by 57.2% (p-value<0.001). Predictors that were only significant for hookworm infection included, at the household level, business ownership and, at the village level, the number of roads. Belonging to a household where the household head owned a business reduced the odds of heavy hookworm infection by 65.4% (p-value<0.001) when compared to subsistence farmers or the unemployed. Having more than two roads, which was a proxy indicator for the spatial spread or low density of households in a village, increased the likelihood of hookworm infections by 93.7% (p-value<0.001).

### *S*. *mansoni* and hookworm infection intensities required for anaemia

Table 3 presents the association of *S*. *mansoni* and hookworm infection intensity with anaemia. The variance components model is provided in Table F (S1 File). Individuals with moderate *S*. *mansoni* infection intensity were 56% more likely (p-value = 0.034) to have anaemia than uninfected individuals. Heavy *S*. *mansoni* infections increased anaemia risk 2.861 fold (p-value<0.001) when compared to participants with no detectable *S*. *mansoni* infection. The probability of anaemia was 65% higher (p-value = 0.008) amongst individuals with heavy hookworm infection intensity when compared with uninfected individuals. These findings were significant despite older age (OR 0.98, p-value<0.001) decreasing the likelihood of anaemia. Only the effect of moderate *S*. *mansoni* infection intensity on anaemia was lost when analyzed against Hb (Table G in S1 File).

**Table 3:** The effect of *S*. *mansoni* and hookworm infection intensities on anaemia.

Variable	Estimate^a^ ^,^ ^b^	Std. Err.	p-value	95% CI	
**Fixed component**					
Light *S*. *mansoni* ^c^	1.063	0.154	0.671	0.801	1.411
Moderate *S*. *mansoni* ^d^	1.562	0.329	0.034	1.034	2.361
Heavy *S*. *mansoni* ^e^	2.861	0.676	<0.001	1.801	4.547
Light hookworm^c^	1.174	0.181	0.298	0.868	1.588
Moderate hookworm^d^	0.870	0.160	0.450	0.607	1.248
Heavy hookworm^e^	1.650	0.313	0.008	1.137	2.393
Age	0.981	0.003	<0.001	0.974	0.987
Female	1.134	0.134	0.286	0.900	1.429
Malaria in past 6 months	0.836	0.109	0.171	0.648	1.080
Business (owns small shop)	0.626	0.159	0.066	0.380	1.031
Fishing (fishermen and fishmongers)	0.708	0.172	0.155	0.440	1.139
Rice farmer	0.828	0.153	0.307	0.577	1.189
School teacher	0.451	0.324	0.267	0.110	1.841
Other employment	1.043	0.253	0.863	0.648	1.678
Lake site only within village (no beach, but small boat landing site on Lake Victoria)	0.760	0.127	0.102	0.548	1.055
3 or more roads within village	0.779	0.129	0.132	0.563	1.078
Constant	1.355	0.255	0.106	0.937	1.960
**Random component**					
Household	0.630	0.230			
Village	0.085	0.054			
**Intraclass correlation**					
Household	0.178	0.049		0.101	0.230
Village	0.021	0.012		0.007	0.064
Obs. 1832	Obs. 1832				
	Crude Global R^2^		0.372		
	Marginal R^2^		0.072		
	Conditional R^2^		0.238		

^a^ General linear latent and mixed model with binomial family.

^b^ Estimate represents odds ratios for fixed components and variances for random components.

^c^ 1–99 eggs per gram (EPG)

^d^ 100–399 EPG

^e^ 400+ EPG

Occupations are represented at the household-level and indicate the employment of the household head.

### Association of *S*. *mansoni* and hookworm coinfection with anaemia

Table 4 presents the association of anaemia with *S*. *mansoni* and hookworm coinfection. Individuals with light, moderate, or heavy coinfection intensity were not significantly at risk of anaemia (p-value>0.05) when compared to participants with no infection or single infections. These results remained robust when coinfection intensities were analyzed against Hb concentration (Table H in S1 File). As a robustness check, a binary indicator was examined for ‘any coinfection’, which was defined as a participant with at least one EPG of *S*. *mansoni* and hookworm. This indicator also was insignificantly related to anaemia and Hb (p-value>0.05) when compared to participants with no infection or single infections (Table I in S1 File).

**Table 4:** Influence of *S*. *mansoni* and hookworm coinfection on anaemia.

Variable	Random intercept model				
	Estimate^a^	Std. Err.	p-value	95% CI	
**Fixed component**					
Light coinfection^b^	1.239	0.225	0.238	0.868	1.770
Moderate coinfection^c^	2.088	0.860	0.074	0.932	4.680
Heavy coinfection^d^	1.863	1.397	0.407	0.428	8.101
Age	0.979	0.003	<0.001	0.973	0.986
Female	1.106	0.129	0.390	0.879	1.390
Malaria in past 6 months	0.821	0.108	0.132	0.634	1.061
Business (owns small shop)	0.675	0.170	0.119	0.412	1.106
Fishing (fishermen and fishmongers)	0.823	0.195	0.412	0.517	1.311
Rice farmer	0.819	0.152	0.280	0.569	1.177
School teacher	0.403	0.290	0.207	0.098	1.653
Other employment	1.039	0.252	0.875	0.646	1.672
Lake site only within village (no beach, but small boat landing site on Lake Victoria)	0.741	0.123	0.070	0.536	1.025
3 or more roads within village	0.780	0.127	0.126	0.568	1.072
Constant	1.662	0.291	0.004	1.179	2.341
**Random component**					
Household	0.674	0.232			
Village	0.080	0.053			
**Intraclass correlation**					
Household	0.186	0.049		0.109	0.300
Village	0.021	0.012		0.007	0.063
Obs. 1832					
Crude Global R^2^	0.404				
Marginal R^2^	0.051				
Conditional R^2^	0.228				

^a^Estimate represents odds ratios for fixed components and variances for random components. General Linear Latent and Mixed Model with Binomial family.

^b^At least 1–99 EPG (eggs per gram) of both *S*. *mansoni* and hookworm.

^c^At least 100–399 EPG of both S. mansoni and hookworm.

^d^At least 400+ EPG of both S. mansoni and hookworm.

The base category for the coinfection variables is no infection and single *S*. *mansoni* and hookworm infections. The variance components (empty) model is presented in Table F in S1 File.

### Anaemia attributable to *S*. *mansoni* and hookworm infections

Table 5 presents the PAF for *S*. *mansoni* and hookworm infection intensities that were found significant for anaemia in the logistic model of Table J (S1 File). Without household and village variation controlled, moderate *S*. *mansoni* was borderline insignificant (p-value = 0.056) against anaemia. In scenario one (Panel 1A), for all individuals, elimination of heavy *S*. *mansoni* infection could reduce anaemia by 3.6% (95% CI 2.1%, 5.0%), whereas elimination of heavy hookworm infections could decrease anaemia by 3.0% (95% CI 1.0%, 5.0%). For adults (excluding pregnant women), only 2.4% (95% CI 1.4%, 3.4%) of anaemia was attributable to *S*. *mansoni* infections. Yet, over 4.5% (95% CI 2.7%, 6.3%) of anaemia for school-aged children, who had the highest infection intensity, and for pregnant women was attributable to *S*. *mansoni* infections. The proportion of anaemia attributable to hookworm infections was greater in age groups with the highest hookworm infection intensities. Adults had a greater fraction of anaemia attributable to hookworm (PAF 4.6%; 95% CI 1.4%, 7.6%) when compared to school-aged children and pregnant women (PAF 1.8%; 95% CI 0.6%, 3%).

**Table 5:** Anaemia attributable to *S*. *mansoni* and hookworm infections.

		A) Elimination strategy^a^				B) Intensity reduction strategy^b^			
	**Scenario**	**PAF** ^c^	**p-value**	**95% CI**		**PAF** ^c^	**p-value**	**95% CI**	
**1) Whole population** (Obs. 1832)	*S*. *mansoni*	0.036	<0.001	0.021	0.050	0.034	<0.001	0.020	0.048
	Hookworm	0.030	0.004	0.010	0.050	0.029	0.004	0.009	0.048
**Population of adults & non-pregnant women only** (Obs. 915)	*S*. *mansoni*	0.024	<0.001	0.014	0.034	0.022	<0.001	0.121	0.033
	Hookworm	0.046	0.004	0.014	0.076	0.044	0.003	0.015	0.072
**1c) Population of school-aged children & pregnant women only** (Obs. 917)	*S*. *mansoni*	0.045	<0.001	0.027	0.063	0.042	<0.001	0.025	0.059
	Hookworm	0.018	0.003	0.006	0.030	0.018	0.003	0.006	0.029
	**Scenario**	**PAFI** ^d^	**p-value**	**95% CI**		**PAFI** ^d^	**p-value**	**95% CI**	
**2) Heavily infected population only** (Obs. 1832)	*S*. *mansoni*	0.320	<0.001	0.215	0.411	0.301	<0.001	0.202	0.389
	Hookworm	0.237	0.002	0.094	0.347	0.222	0.001	0.092	0.333
**Heavily infected adults & non-pregnant women only** (Obs. 915)	*S*. *mansoni*	0.370	<0.001	0.252	0.470	0.351	<0.001	0.231	0.453
	Hookworm	0.249	0.002	0.100	0.374	0.241	0.001	0.104	0.357
**Heavily infected school-aged children & pregnant women only** (Obs. 917)	*S*. *mansoni*	0.304	<0.001	0.203	0.392	0.285	<0.001	0.190	0.369
	Hookworm	0.202	0.001	0.085	0.304	0.193	0.001	0.083	0.290

^a^Model used in Table J in S1 File. The elimination strategy concerns eliminating, reducing to zero, hookworm or *S*.*mansoni* infection intensity.

^b^PAF is the population attributable fraction amongst uninfected and infected individuals.

^c^Model used in Table K in S1 File. The intensity reduction strategy concerns reducing hookworm or *S*. *mansoni* infection to uninfected/low/moderate (<400 EPG).

^d^PAFI is the population attributable fraction amongst only infected individuals, i.e. in a subpopulation with heavy hookworm or *S*. *mansoni* infection (400+ EPG).

In Panel 2, two subsets of the population with either heavy *S*. *mansoni* or heavy hookworm infections were used to calculate the population attributable fractions amongst the infected (PAFI). The elimination of *S*. *mansoni* in individuals with heavy *S*. *mansoni* infection could reduce anaemia by 32.0% (95% CI 21.5%, 41.1%). Hookworm elimination amongst people with heavy hookworm infection could decrease anaemia by 23.7% (95% CI 9.4%, 34.7%). The effects of eliminating infections were further examined by age for individuals with heavy infection intensities. Heavily infected adults, excluding pregnant women, had a larger fraction of anaemia attributable to both helminth infections when compared to heavily infected school-aged children and pregnant women. Curing heavily infected, non-pregnant adults could decrease 37% (95% CI 25.2%, 47%) and 24.9% (95% CI 10%, 37.4%) of anaemia attributable, respectively, to heavy *S*. *mansoni* and hookworm infections. Eliminating infections in heavily infected school-aged children and pregnant women may reduce 30.4% (95% CI 20.3%, 39.2%) and 20.2% (95% CI 8.5%, 30.4%) of anaemia due to heavy *S*. *mansoni* and hookworm infections, respectively.

In scenario two, the PAF was calculated using a strategy of only reducing infection intensity as opposed to curing individuals. Covariates were accounted for and a model was used similar to that used in Table J (S1 File) except indicators were excluded for low and moderate *S*. *mansoni* and hookworm infections (Table K in S1 File). This analysis presented the potential impact of reducing heavy *S*. *mansoni* infections to uninfected/low/moderate *S*. *mansoni* infections and reducing heavy hookworm infections to low/uninfected/moderate hookworm infections. Decreasing intensity would have an impact on anaemia for the full sample, school-aged children and pregnant women, or only adults (excluding pregnant women) that is comparable to the elimination of these infections from these groups. PAFs and PAFIs only differed, respectively, by 0.01–0.3% and 0.8–1.9% (Panels 1B, 2B in Table 5).

## Discussion

With an increase in the number of anaemia cases from the year 1990 to 2010 in SSA [1], a better understanding is needed of how to simplify monitoring and treatment of the multiple causes of anaemia. In this paper, we assessed the associations of *S*. *mansoni* and hookworm infections with anaemia. A community-based sample was used of 1,832 individuals aged 5–90 years across 30 villages in Uganda. When examined against age, peak infection intensities coincided with the highest prevalence of anaemia. The heaviest intensity of *S*. *mansoni* was in children and the highest intensity of hookworm was in the elderly. Predictors of infection risk including age, gender, occupation, and village-level factors were significant for only one infection or opposite in direction for *S*. *mansoni* and hookworm. Differences in *S*. *mansoni* and hookworm risk factors have been shown in studies of single infections [47, 48]. To our knowledge this study is the first to show that *S*. *mansoni* and hookworm infections influence anaemia in separate groups of individuals within one study setting. In community-based samples, guidelines to target individuals requiring treatment for anaemia due to *S*. *mansoni* or hookworm infections must delineate separate profiles for each infection. Yet, community-based sampling can be used to capture diverse causes of anaemia due to both intestinal helminths.

Our study was able to identify the relative contribution of *S*. *mansoni* and hookworm infections to anaemia despite potential confounders of malnutrition and malaria. Malnutrition is an important aspect of anaemia risk [12]. For malnutrition to be a confounder in our analysis, it must have an individual-level effect on anaemia risk. The multilevel model in this study accounted for latent variation in anaemia attributable to household and village variance. Accordingly, important unobserved differences between households or communities for malnutrition were considered in these latent factors. For example, household factors that affect anaemia may include maternal care for children, household diet and meals, or socioeconomic factors whereas community factors may include sanitation infrastructure or physical proximity to a health centre [49, 50]. At the individual level, Pullan et al. finds significant associations of *S*. *mansoni* and hookworm infections with anaemia after controlling for malnutrition in schoolchildren in Kenya [22].

Self-reported malaria, which indicated if study participants received treatment for malaria in the six months preceding the study, was uncorrelated with anaemia. For malaria to be a confounder in our study, two issues must be observed. Formal diagnoses of malaria must conflict with self-reported treatment and a specific set of individuals must be coinfected, i.e. individuals with heavy *S*. *mansoni* or heavy hookworm infection intensities. Coinfections of *P*. *falciparum* and *S*. *mansoni* or hookworm have been shown in SSA to increase anaemia prevalence in children when compared to single *S*. *mansoni* or hookworm infections [51, 52]. Such coinfections have been shown to have a prevalence of 26.47% in children aged 10–14 years in our study area and are a limitation of this study [53]. Another factor to consider is if malaria prevalence decreases with the distance to freshwater bodies, as was the case with *S*. *mansoni* infections. However, this confounder is unlikely in our study setting since individuals within the same village were only 2.1% more likely to have anaemia when compared to individuals of other villages (ICC, Table 3). Also, the presence of swamps in a village and the distance of the village centre to Lake Victoria was investigated and found insignificant (p-value>0.05) for anaemia risk (Table 3, Table E in S1 File). Concerning other coinfections, additional intestinal helminths were directly examined as potential predictors of anaemia. The infection prevalence of trichuriasis was 1.04% and ascariasis was 0.11% in our study villages, but no associations with anaemia were found (Spearman correlations: Trichuriasis rho 0.028, p-value = 0.234; Ascariasis rho -0.030, p-value 0.207).

The relationships of *S*. *mansoni* and hookworm infections with anaemia were intensity-dependent; heavy infection intensities (400+ EPG) significantly increased anaemia risk. Individuals with heavy *S*. *mansoni* were 2.86 times more likely to have anaemia than uninfected participants. This effect amongst our community-based sample is considerably larger than the influence of heavy *S*. *mansoni* infection in individuals sampled from Ugandan primary schools, who had increased anaemia risk of 1.57 fold [19]. Further work is needed to identify if community-based samples best include children at the highest risk of anaemia from *S*. *mansoni* infection. Community-based samples, such as our study, include many non-enrolled children and heavy infections may be concentrated in this group.

Although beyond the scope of this study, there is a need to identify the major mechanism of *S*. *mansoni* infection that causes anaemia. *S*. *mansoni* may be in direct competition with the human host for iron in the intestinal and blood stages of the parasite [54]. Alternatively, blood loss could occur from intestinal bleeding when eggs need to traverse the epithelial, mucosal barrier [10, 15, 55]. If the intestinal epithelium is compromised then host intestinal iron absorption can be impaired [55]. Additionally, if severe *S*. *mansoni* infection elevates markers of inflammation such as Interleukin 6, then a feedback mechanism could occur that inhibits host intestinal iron absorption through up-regulation of hepcidin [56]. In addition to Hb counts, to address these possible causes, future studies must measure occult blood and indicators of iron metabolism such as serum ferritin and soluble transferrin receptors [15, 56].

Heavy hookworm infections increased anaemia risk 1.65 fold when compared to people with no detectable hookworm infection. This estimate concurs with existing knowledge of moderate and heavy hookworm infection intensity and anaemia [7, 8]. The effect of hookworm infection may appear less than the influence of *S*. *mansoni* infection on anaemia. However, in this study, heavy hookworm infection intensity was classified as the top 10^th^ percentile of hookworm infections (400+ EPG). Although 400 or more EPG is the WHO classification of heavy *S*. *mansoni* infection, this intensity is considered by the WHO as a light hookworm infection [33]. Pullen et al. [22] shows very light hookworm infections in Kenyan schoolchildren (100 EPG) are associated with anaemia. In this paper, by assessing entire communities, a sensitive detection was possible of the relative contribution of hookworm infection to anaemia for every age group. We showed that the elderly are vulnerable to anaemia with only light hookworm infections. With morbidity detectable from only light hookworm infections, future research is warranted to identify the hookworm species present in the study area. Hookworm virulence can vary depending on the species. Albonico et al. has shown that *Ancylostoma duodenale*, although less prevalent than *Necator americanus*, can cause greater iron-deficiency anaemia [57].

*S*. *mansoni* and hookworm coinfections were insignificant for anaemia when compared to no infection or single infections. This result is consistent with studies in Uganda and Rwanda that assessed *S*. *mansoni* and hookworm coinfections in children [16, 27]. Coinfection was insignificant in our study because only heavy infection intensity was associated with anaemia, and heavy *S*. *mansoni* and heavy hookworm infections did not overlap by age, socioeconomic, or environmental characteristics. If repeated in other settings, this finding can provide explanations for why other studies in SSA [16, 27] also have failed to find associations between anaemia and coinfections of *S*. *mansoni* and hookworm.

Treatment of anaemia must be made a priority for individuals with heavy *S*. *mansoni* and heavy hookworm infections. A large proportion of anaemia amongst infected individuals was attributable to intestinal helminths. *S*. *mansoni* explained 32.0% of anaemia in individuals with heavy *S*. *mansoni* infection intensity. For people with heavy hookworm infections, 23.7% of anaemia could be attributed to hookworm infections. When the anaemia attributable to helminth infections was further analyzed by age, the proportion of anaemia due to *S*. *mansoni* or hookworm infections was greater in sub-groups with higher average infection intensities. Children had a greater fraction of anaemia attributable to *S*. *mansoni* infections than adults and non-pregnant women. However, adults (excluding pregnant women) had a greater proportion of anaemia due to hookworm infections when compared to school-aged children and pregnant women. These findings concur with our results that show anaemia prevalence peaks in age groups that have the highest infection intensities. What is striking about our results is that when only heavily infected individuals were examined by age, the anaemia attributable to both *S*. *mansoni* and hookworm infections was highest amongst adults. This finding raises an important question for future studies. Do children have more competing, multifactorial causes of anaemia than adults? If so, how does competition amongst causes of anaemia affect the morbidity caused by helminth infections? Most importantly, amongst heavily infected individuals, adjusted PAF analysis suggested that reducing infection intensity would have an impact on anaemia of only 1–2% lower than efforts to eliminate heavy *S*. *mansoni* or hookworm infections. Hence, anthelminthic treatment, which is the only strategy widely implemented for controlling *S*. *mansoni* and hookworm infection intensities, is likely the most effective method to decrease anaemia attributable to these infections [19, 25, 26].

Preventive chemotherapies are widely distributed through MDA to treat human helminthiases and have been administered annually since 2003 in our study area [29]. Yet, within the context of MDA, we found that widespread morbidity persists due to intestinal helminths. With increased funding commitments and drug donations [58] as well as programmes progressing from infection control to elimination [59], there is an imminent need to expand treatment coverage with PCs for communitywide anaemia. Schoolchildren, who bear the greatest burden of anaemia [2], must remain a priority for the WHO. In addition, for areas endemic with schistosomiasis and hookworm infections, our study directly appeals to the WHO to establish guidelines to include adults for treatment in helminth control programmes [33]. Adult eligibility for treatment is limited. Praziquantel is only available for adults in high-risk communities, which include areas with greater than 50% schistosomiasis infection prevalence in schoolchildren. For hookworm infections, adults mainly receive indirect treatment. When communities are targeted for lymphatic filariasis, all individuals aged five years and older are eligible for a package of albendazole and ivermectin. Only women of childbearing age and a few occupations including tea pickers and miners are eligible for hookworm treatment [33]. Our study suggests that guidelines for treating hookworm infection should not be limited to children and pregnant or potentially pregnant women. We demonstrated that adults exhibit serious morbidity associated with helminth infections. The availability of treatment for the elderly is of particular concern. By harbouring the heaviest hookworm infections, the elderly are a main reservoir of reinfection for not only schoolchildren, but also entire communities. If community-based epidemiological surveys are repeated in other countries, our findings can help consolidate WHO guidelines for use of preventive chemotherapies in SSA to treat anaemia caused by intestinal helminths.

## Supporting Information

S1 File Supplementary information containing 11 tables. Table A: Descriptive statistics of binary variables. Table B: Descriptive statistics of continuous variables. Table C: *S*. *mansoni* and hookworm intensities and haemoglobin by age group. Table D: *S*. *mansoni*, hookworm, and anaemia prevalence by age group. Table E: Univariate models for candidate variables in anaemia logistic model. Table F: Multilevel model on dependent variable of anaemia. Table G: Multilevel model on dependent variable of Hb. Table H: Any *S*. *mansoni* and hookworm infections on dependent variable of anaemia. Table I: Heavy *S*. *mansoni* and hookworm infections on dependent variable of anaemia. Table J: Any *S*. *mansoni* and hookworm infections on dependent variable of anaemia. Table K: Heavy *S*. *mansoni* and hookworm infections on dependent variable of anaemia.
(DOCX)

S1 Dataset Study participant data. (XLS)

S1 Checklist STROBE checklist. (DOC)
