# Correction: Influence of *Schistosoma mansoni* and Hookworm Infection Intensities on Anaemia in Ugandan Villages

**DOI:** 10.1371/journal.pntd.0004287

**Published:** 2015-12-04

**Authors:** Goylette F. Chami, Alan Fenwick, Erwin Bulte, Andreas A. Kontoleon, Narcis B. Kabatereine, Edridah M. Tukahebwa, David W. Dunne

The affiliation of authors 1, 3 and 4 is spelled incorrectly. The correct affiliation is Department of Land Economy, University of Cambridge, Cambridge, United Kingdom
