# Supplementary material for: Influence of Schistosoma mansoni and Hookworm Infection Intensities on Anaemia in Ugandan Villages
Source: PLoS Negl Trop Dis. 2015 Oct 29;9(10):e0004193. doi: 10.1371/journal.pntd.0004193 (PMC4626098; doi:10.1371/journal.pntd.0004193)
Supplement: S1 File — Table A: Descriptive statistics of binary variables. Table B: Descriptive statistics of continuous variables. Table C: S. mansoni and hookworm intensities and haemoglobin by age group. Table D: S. mansoni, hookworm, and anaemia prevalence by age group. Table E: Univariate models for candidate variables in anaemia logistic model. Table F: Multilevel model on dependent variable of anaemia. Table G: Multilevel model on dependent variable of Hb. Table H: Any S. mansoni and hookworm infections on dependent variable of anaemia. Table I: Heavy S. mansoni and hookworm infections on dependent variable of anaemia. Table J: Any S. mansoni and hookworm infections on dependent variable of anaemia. Table K: Heavy S. mansoni and hookworm infections on dependent variable of anaemia. (DOCX) [file pntd.0004193.s001.docx]

**Supplementary information for:** Influence of *Schistosoma mansoni* and hookworm infection intensities on anaemia in Ugandan villages

Goylette F. Chami, Alan Fenwick, Erwin Bulte, Andreas A. Kontoleon, Narcis B. Kabatereine, Edridah M. Tukahebwa, David W. Dunne

Table of Contents

Table A: Descriptive statistics of binary variables 2

Table B: Descriptive statistics of continuous variables 2

Table C: *S. mansoni* and hookworm intensities and haemoglobin by age group 3

Table D: *S. mansoni*, hookworm, coinfection, and anaemia prevalence by age group 3

Table E: Univariate models for candidate variables in anaemia logistic model 3

Table F: Multilevel model on dependent variable of anaemia 4

Table G: Multilevel model on dependent variable of haemoglobin g/L 5

Table H: Influence of *S. mansoni* and hookworm coinfection on haemoglobin g/L 6

Table I: Influence of any *S. mansoni* and hookworm coinfection on anaemia and haemoglobin g/L 7

Table J: Any *S. mansoni* and hookworm infections on dependent variable of anaemia 8

Table K: Heavy *S. mansoni* and hookworm infections on dependent variable of anaemia 8

References 9

## Table A: Descriptive statistics of binary variables

| **Variable** | **Percent** | **Frequency** |
| --- | --- | --- |
| Female^a^ | 59.72 | 1094/1832 |
| *S. mansoni* prevalence | 36.41 | 667/1832 |
| Light *S. mansoni* prevalence | 20.52 | 376/1832 |
| Moderate *S. mansoni* prevalence | 8.30 | 152/1832 |
| Heavy *S. mansoni* prevalence | 7.59 | 139/1832 |
| Hookworm prevalence | 40.45 | 741/1832 |
| Light hookworm prevalence | 17.30 | 317/1832 |
| Moderate hookworm prevalence | 11.68 | 214/1832 |
| Heavy hookworm prevalence | 11.46 | 210/1832 |
| Anaemia | 44.38 | 813/1832 |
| Malaria in past 6 months | 45.31 | 830/1832 |
| Business (owns small shop)^b^ | 6.33 | 116/1832 |
| Fishing (fishermen and fishmongers)^b^ | 7.31 | 134/1832 |
| Subsistence farmer^b^ | 64.41 | 1180/1832 |
| Rice farmer^b^ | 14.52 | 266/1832 |
| School teacher^b^ | 0.76 | 14/1832 |
| Other employment^b^ | 6.66 | 122/1832 |
| Rice paddy (large rice farm) within village^c^ | 76.75 | 1406/1832 |
| Beach on Lake Victoria within village^c^ | 23.25 | 426/1832 |
| Lake site only within village (no beach, but small boat landing site on Lake Victoria)^c^ | 36.68 | 672/1832 |
| Village centre more than 0.50 km to Lake Victoria^c^ | 63.43 | 1162/1832 |
| 3 or more roads within village | 43.45 | 796/1832 |

^a^The female to male ratio of participants was 1.48 (1094/738).

^b^ Household-level variable, which represents occupation of household head.

^c^ Village-level variable.

## Table B: Descriptive statistics of continuous variables

| **Variable** | **Obs.** | **Mean** | **Std. Dev.** | **Min** | **Max** |
| --- | --- | --- | --- | --- | --- |
| Age | 1832 | 24.309 | 16.901 | 4 | 90 |
| *S. mansoni* EPG | 1832 | 144.865 | 674.788 | 0 | 11964 |
| Hookworm EPG | 1832 | 299.122 | 1236.446 | 0 | 24000 |
| LN (*S. mansoni* EPG +1) | 1832 | 1.654 | 2.391 | 0 | 9.390 |
| LN (Hookworm EPG + 1) | 1832 | 2.051 | 2.714 | 0 | 10.086 |
| Haemoglobin g/L | 1832 | 121.018 | 17.572 | 11 | 200 |
| LN (Total houses in village) | 1832 | 5.420 | 0.491 | 4.331 | 6.589 |

EPG represents eggs per gram and LN represents natural log.

## Table C: *S. mansoni* and hookworm intensities and haemoglobin by age group

| **A) *S. mansoni* eggs per gram (EPG)** | | |  |  |  |
| --- | --- | --- | --- | --- | --- |
| **Age group** | **Obs.** | **Mean** | **Std. Dev.** | **Min.** | **Max.** |
| 5-9 | 438 | 218.027 | 836.127 | 0 | 11232 |
| 10-15 | 477 | 214.491 | 801.654 | 0 | 8400 |
| 16-40 | 587 | 81.935 | 550.739 | 0 | 11964 |
| 40-60 | 289 | 64.484 | 348.003 | 0 | 4392 |
| 61+ | 41 | 20.780 | 73.290 | 0 | 420 |
|  |  |  |  |  |  |
| **B) Hookworm EPG** |  |  |  |  |  |
| **Age group** | **Obs.** | **Mean** | **Std. Dev.** | **Min.** | **Max.** |
| 5-9 | 438 | 133.863 | 917.574 | 0 | 14316 |
| 10-15 | 477 | 216.000 | 754.445 | 0 | 6684 |
| 16-40 | 587 | 369.547 | 1542.549 | 0 | 24000 |
| 40-60 | 289 | 506.491 | 1473.707 | 0 | 10008 |
| 61+ | 41 | 561.659 | 1636.332 | 0 | 9696 |
|  |  |  |  |  |  |
| **C) Haemoglobin g/L** |  |  |  |  |  |
| **Age group** | **Obs.** | **Mean** | **Std. Dev.** | **Min.** | **Max.** |
| 5-9 | 438 | 111.548 | 14.290 | 37 | 160 |
| 10-15 | 477 | 117.767 | 14.459 | 11 | 165 |
| 16-40 | 587 | 126.082 | 18.326 | 59 | 200 |
| 40-60 | 289 | 129.180 | 16.635 | 79 | 179 |
| 61+ | 41 | 129.951 | 21.353 | 78 | 188 |

## Table D: *S. mansoni*, hookworm, coinfection, and anaemia prevalence by age group

|  | ***S. mansoni* prevalence** | | **Hookworm prevalence** | | **Coinfection prevalence** | | **Anaemia prevalence** | |
| --- | --- | --- | --- | --- | --- | --- | --- | --- |
| **Age group** | **Percent** | **Frequency** | **Percent** | **Frequency** | **Percent** | **Frequency** | **Percent** | **Frequency** |
| 5-9 | 41.78 | 183/438 | 28.77 | 126/438 | 10.5 | 46/438 | 56.39 | 247/438 |
| 10-15 | 44.65 | 213/477 | 41.3 | 197/477 | 16.35 | 78/477 | 48.22 | 230/477 |
| 16-40 | 32.2 | 189/587 | 45.83 | 269/587 | 14.82 | 87/587 | 37.31 | 219/587 |
| 40-60 | 25.95 | 75/289 | 43.6 | 126/289 | 8.3 | 24/289 | 34.6 | 100/289 |
| 61+ | 17.07 | 7/41 | 56.1 | 23/41 | 12.2 | 5/41 | 41.46 | 17/41 |

Coinfection is at least one egg per gram of both *S. mansoni* and hookworm infections.

## Table E: Univariate models for candidate variables in anaemia logistic model

| **Variable** | **Odds ratio^a^** | **Std. Err.** | **p-value**^b^ | **95% CI** | |
| --- | --- | --- | --- | --- | --- |
| Age grand-mean-centered squared | 1.000 | 0.000 | 0.353 | 1.000 | 1.000 |
| Malaria in past 6 months | 0.678 | 0.064 | <0.001 | 0.563 | 0.817 |
| Rice paddy (large rice farm) within village | 0.964 | 0.107 | 0.743 | 0.775 | 1.199 |
| Beach on Lake Victoria within village | 1.131 | 0.126 | 0.269 | 0.910 | 1.406 |
| Lake site only within village (no beach, but small boat landing site on Lake Victoria) | 0.778 | 0.076 | 0.010 | 0.642 | 0.943 |
| Village centre more than 0.50 km to Lake Victoria | 0.956 | 0.093 | 0.649 | 0.790 | 1.158 |
| 3 or more roads within village | 0.803 | 0.076 | 0.021 | 0.667 | 0.968 |
| Natural log of total houses in village | 1.100 | 0.105 | 0.319 | 0.912 | 1.327 |
| Obs. 1832 |  |  |  |  |  |
| ^a^Univariate logistic models |  |  |  |  |  |
| ^b^P-value of the likelihood ratio test against empty model | | | |  |  |

## Table F: Multilevel model on dependent variable of anaemia

|  | **A) Empty/Variance components model**^a^ | | | | | **B) Random intercept model** | | | | |
| --- | --- | --- | --- | --- | --- | --- | --- | --- | --- | --- |
| **Variable** | **Estimate** | **Std. Err.** | **p-value** | **95% CI** | | **Estimate** | **Std. Err.** | **p-value** | **95% CI** | |
| **Fixed component** |  |  |  |  |  | **Fixed component** | | |  |  |
| *S. mansoni* 1-99 EPG |  |  |  |  |  | 1.063 | 0.154 | 0.671 | 0.801 | 1.411 |
| *S. mansoni* 100-399 EPG | |  |  |  |  | 1.562 | 0.329 | 0.034 | 1.034 | 2.361 |
| *S. mansoni* 400+ EPG |  |  |  |  |  | 2.861 | 0.676 | <0.001 | 1.801 | 4.547 |
| Hookworm 1-99 EPG |  |  |  |  |  | 1.174 | 0.181 | 0.298 | 0.868 | 1.588 |
| Hookworm 100-399 EPG | |  |  |  |  | 0.870 | 0.160 | 0.450 | 0.607 | 1.248 |
| Hookworm 400+ EPG |  |  |  |  |  | 1.650 | 0.313 | 0.008 | 1.137 | 2.393 |
| Age |  |  |  |  |  | 0.981 | 0.003 | <0.001 | 0.974 | 0.987 |
| Female |  |  |  |  |  | 1.134 | 0.134 | 0.286 | 0.900 | 1.429 |
| Malaria in past 6 months |  |  |  |  |  | 0.836 | 0.109 | 0.171 | 0.648 | 1.080 |
| Business (owns small shop) |  |  |  |  |  | 0.626 | 0.159 | 0.066 | 0.380 | 1.031 |
| Fishing (fishermen and fishmongers) |  |  |  |  |  | 0.708 | 0.172 | 0.155 | 0.440 | 1.139 |
| Rice farmer |  |  |  |  |  | 0.828 | 0.153 | 0.307 | 0.577 | 1.189 |
| School teacher |  |  |  |  |  | 0.451 | 0.324 | 0.267 | 0.110 | 1.841 |
| Other employment |  |  |  |  |  | 1.043 | 0.253 | 0.863 | 0.648 | 1.678 |
| Lake site only within village (no beach, but small boat landing site on Lake Victoria) |  |  |  |  |  | 0.760 | 0.127 | 0.102 | 0.548 | 1.055 |
| 3 or more roads within village |  |  |  |  |  | 0.779 | 0.129 | 0.132 | 0.563 | 1.078 |
| Constant | 0.769 | 0.073 | 0.006 | 0.639 | 0.926 | 1.355 | 0.255 | 0.106 | 0.937 | 1.960 |
|  |  |  |  |  |  |  |  |  |  |  |
| **Random component** |  |  |  |  |  | **Random component** | | |  |  |
| Household | 0.556 | 0.211 |  |  |  | 0.630 | 0.230 |  |  |  |
| Village | 0.171 | 0.071 |  |  |  | 0.085 | 0.054 |  |  |  |
| **Intraclass correlation** |  |  |  |  |  |  |  |  |  |  |
| Household | 0.181 | 0.047 |  | 0.106 | 0.292 | 0.178 | 0.049 |  | 0.101 | 0.230 |
| Village | 0.043 | 0.017 |  | 0.019 | 0.090 | 0.021 | 0.012 |  | 0.007 | 0.064 |
| Obs. 1832 |  |  |  |  |  | Obs. 1832 | |  |  |  |
|  |  |  |  |  |  | Crude Global R^2^ | | 0.372 |  |  |
|  |  |  |  |  |  | Marginal R^2^ | | 0.072 |  |  |
|  |  |  |  |  |  | Conditional R^2^ | | 0.238 |  |  |
| ^a^Estimate represents odds ratios for fixed components and variances for random components. | | | | | | | | | |  |
| General Linear Latent and Mixed Model with Binomial family.  EPG represents eggs per gram. Occupations are represented at the household-level and indicate the employment of the household head. | | | | | |  |  |  |  |  |

The explanation of this model can be found in the methods section of the main text. We include this model in the supplement to present the initial empty variance components model. The empty model excludes fixed effects.

## Table G: Multilevel model on dependent variable of haemoglobin g/L

|  | **A) Empty/Variance components model**^a^ | | | | | **B) Random intercept model** | | | | |
| --- | --- | --- | --- | --- | --- | --- | --- | --- | --- | --- |
| **Variable** | **Estimate** | **Std. Err.** | **p-value** | **95% CI** | | **Estimate** | **Std. Err.** | **p-value** | **95% CI** | |
| **Fixed component** | |  |  |  |  | **Fixed component** | | |  |  |
| *S. mansoni* 1-99 EPG | |  |  |  |  | -0.707 | 0.958 | 0.461 | -2.585 | 1.171 |
| *S. mansoni* 100-399 EPG | |  |  |  |  | -0.540 | 1.405 | 0.701 | -3.293 | 2.213 |
| *S. mansoni* 400+ EPG | |  |  |  |  | -5.400 | 1.516 | <0.001 | -8.372 | -2.429 |
| Hookworm 1-99 EPG | |  |  |  |  | -1.205 | 1.017 | 0.236 | -3.198 | 0.788 |
| Hookworm 100-399 EPG | |  |  |  |  | 0.471 | 1.197 | 0.694 | -1.875 | 2.818 |
| Hookworm 400+ EPG | |  |  |  |  | -5.460 | 1.261 | <0.001 | -7.931 | -2.989 |
| Age |  |  |  |  |  | 0.393 | 0.022 | <0.001 | 0.351 | 0.436 |
| Female |  |  |  |  |  | -5.972 | 0.774 | <0.001 | -7.488 | -4.455 |
| Malaria in past 6 months | |  |  |  |  | 0.419 | 0.854 | 0.624 | -1.255 | 2.092 |
| Business (owns small shop) |  |  |  |  |  | 3.857 | 1.620 | 0.017 | 0.681 | 7.033 |
| Fishing (fishermen and fishmongers) |  |  |  |  |  | 4.813 | 1.573 | 0.002 | 1.730 | 7.896 |
| Rice farmer |  |  |  |  |  | 2.231 | 1.171 | 0.057 | -0.065 | 4.527 |
| School teacher |  |  |  |  |  | 9.201 | 4.352 | 0.035 | 0.670 | 17.731 |
| Other employment | |  |  |  |  | 0.259 | 1.569 | 0.869 | -2.816 | 3.334 |
| Lake site only within village (no beach, but small boat landing site on Lake Victoria) |  |  |  |  |  | 1.919 | 1.316 | 0.145 | -0.661 | 4.498 |
| 3 or more roads within village |  |  |  |  |  | 1.743 | 1.296 | 0.179 | -0.798 | 4.284 |
| Constant | 121.019 | 0.762 | <0.001 | 119.525 | 122.512 | 113.741 | 1.361 | <0.001 | 111.073 | 116.409 |
|  |  |  |  |  |  |  |  |  |  |  |
| **Random component** | |  |  |  |  | **Random component** | | |  |  |
| Individual | 296.048 | 9.863 |  |  |  | 222.537 | 10.457 |  |  |  |
| Household | <0.0001 | 0.050 |  |  |  | 16.610 | 8.080 |  |  |  |
| Village | 12.563 | 4.498 |  |  |  | 7.541 | 3.224 |  |  |  |
| **Intraclass correlation** | |  |  |  |  |  |  |  |  |  |
| Household | 0.041 | 0.014 |  | 0.020 | 0.079 | 0.098 | 0.034 |  | 0.049 | 0.188 |
| Village | 0.041 | 0.014 |  | 0.020 | 0.079 | 0.032 | 0.013 |  | 0.014 | 0.069 |
| Obs. 1832 |  |  |  |  |  | Obs. 1832 | |  |  |  |
|  |  |  |  |  |  | Crude Global R^2^ | | 0.317 |  |  |
|  |  |  |  |  |  | Marginal R^2^ | | 0.207 |  |  |
|  |  |  |  |  |  | Conditional R^2^ | | 0.290 |  |  |
| ^a^Estimate represents coefficients for fixed components and variances for random components. | | | | | | | | | |  |
| General Linear Latent and Mixed Model with Gaussian family. Occupations are represented at the household-level and indicate the employment of the household head. | | | | | |  |  |  |  |  |

This model is similar to the anaemia model except haemoglobin (Hb) is the dependent variable. Hb is represented as a continuous variable in grams/Litre. The multilevel model is specified with a Gaussian family. We present this multilevel analysis of Hb as a robustness check to the anaemia model and to provide an additional measure for comparisons to other published studies.

## Table H: Influence of *S. mansoni* and hookworm coinfection on haemoglobin g/L

|  | **Random intercept model** | | | | |
| --- | --- | --- | --- | --- | --- |
| **Variable** | **Estimate**^a^ | **Std. Err.** | **p-value** | **95% CI** | |
| **Fixed component** |  | | | | |
| Light coinfection^b^ | -1.545 | 1.216 | 0.204 | -3.929 | 0.839 |
| Moderate coinfection^c^ | -2.742 | 2.705 | 0.311 | -8.045 | 2.560 |
| Heavy coinfection^d^ | -2.606 | 4.970 | 0.600 | -12.348 | 7.135 |
| Age | 0.390 | 0.021 | <0.001 | 0.348 | 0.431 |
| Female | -5.959 | 0.775 | <0.001 | -7.477 | -4.440 |
| Malaria in past 6 months | 0.553 | 0.866 | 0.523 | -1.145 | 2.251 |
| Business (owns small shop) | 3.599 | 1.638 | 0.028 | 0.387 | 6.810 |
| Fishing (fishermen and fishmongers) | 4.333 | 1.578 | 0.006 | 1.241 | 7.425 |
| Rice farmer | 2.297 | 1.190 | 0.054 | -0.036 | 4.630 |
| School teacher | 10.005 | 4.419 | 0.024 | 1.343 | 18.667 |
| Other employment | 0.295 | 1.595 | 0.853 | -2.831 | 3.421 |
| Lake site only within village (no beach, but small boat landing site on Lake Victoria) | 1.943 | 1.317 | 0.140 | -0.638 | 4.524 |
| 3 or more roads within village | 1.434 | 1.290 | 0.266 | -1.094 | 3.963 |
| Constant | 112.779 | 1.289 | <0.001 | 110.253 | 115.305 |
|  |  |  |  |  |  |
| **Random component** | | | | | |
| Individual | 222.927 | 10.463 |  |  |  |
| Household | 20.833 | 8.221 |  |  |  |
| Village | 7.442 | 3.264 |  |  |  |
| **Intraclass correlation** |  |  |  |  |  |
| Household | 0.113 | 0.034 |  | 0.061 | 0.198 |
| Village | 0.031 | 0.013 |  | 0.014 | 0.068 |
| Obs. 1832 |  |  |  |  |  |
| Crude Global R^2^ | 0.332 |  |  |  |  |
| Marginal R^2^ | 0.193 |  |  |  |  |
| Conditional R^2^ | 0.292 |  |  |  |  |

^a^Estimate represents odds ratios for fixed components and variances for random components. General Linear Latent and Mixed Model with Gaussian family.

^b^At least 1-99 EPG (eggs per gram) of both *S. mansoni* and hookworm.

^c^At least 100-399 EPG of both S. mansoni and hookworm.

^d^At least 400+ EPG of both S. mansoni and hookworm.

The base category for the coinfection variables is no infection and single *S. mansoni* and hookworm infections. The variance components model is presented in Table S7.

## Table I: Influence of any *S. mansoni* and hookworm coinfection on anaemia and haemoglobin g/L

|  | **A) Random intercept model with dependent variable of anaemia** | | | | | **B) Random intercept model with dependent variable of haemoglobin g/L** | | | | |
| --- | --- | --- | --- | --- | --- | --- | --- | --- | --- | --- |
| **Variable** | **Estimate**^a^ | **Std. Err.** | **p-value** | **95% CI** | | **Estimate**^a^ | **Std. Err.** | **p-value** | **95% CI** | |
| **Fixed component** |  | | |  |  |  |  |  |  |  |
| Any coinfection | 1.358 | 0.226 | 0.066 | 0.980 | 1.883 | -1.760 | 1.118 | 0.116 | -3.952 | 0.432 |
| Age | 0.980 | 0.003 | <0.001 | 0.973 | 0.986 | 0.389 | 0.021 | <0.001 | 0.348 | 0.431 |
| Female | 1.102 | 0.129 | 0.403 | 0.877 | 1.386 | -5.953 | 0.775 | <0.001 | -7.472 | -4.434 |
| Malaria in past 6 months | 0.820 | 0.108 | 0.130 | 0.634 | 1.060 | 0.555 | 0.866 | 0.522 | -1.143 | 2.253 |
| Business (owns small shop) | 0.677 | 0.170 | 0.121 | 0.414 | 1.108 | 3.594 | 1.638 | 0.028 | 0.383 | 6.804 |
| Fishing (fishermen and fishmongers) | 0.830 | 0.197 | 0.431 | 0.521 | 1.320 | 4.316 | 1.577 | 0.006 | 1.225 | 7.407 |
| Rice farmer | 0.815 | 0.151 | 0.269 | 0.567 | 1.171 | 2.306 | 1.189 | 0.052 | -0.025 | 4.638 |
| School teacher | 0.400 | 0.287 | 0.202 | 0.098 | 1.636 | 10.027 | 4.418 | 0.023 | 1.367 | 18.687 |
| Other employment | 1.031 | 0.250 | 0.900 | 0.641 | 1.658 | 0.317 | 1.594 | 0.842 | -2.806 | 3.441 |
| Lake site only within village (no beach, but small boat landing site on Lake Victoria) | 0.739 | 0.123 | 0.068 | 0.534 | 1.023 | 1.950 | 1.318 | 0.139 | -0.634 | 4.535 |
| 3 or more roads within village | 0.779 | 0.127 | 0.124 | 0.567 | 1.071 | 1.439 | 1.291 | 0.265 | -1.091 | 3.969 |
| Constant | 1.664 | 0.291 | 0.004 | 1.181 | 2.346 | 112.773 | 1.290 | <0.001 | 110.245 | 115.301 |
|  |  |  |  |  |  |  |  |  |  |  |
| **Random component** |  |  |  |  |  |  |  |  |  |  |
| Individual |  |  |  |  |  | 223.010 | 10.460 |  |  |  |
| Household | 0.666 | 0.230 |  |  |  | 20.759 | 8.209 |  |  |  |
| Village | 0.081 | 0.054 |  |  |  | 7.475 | 3.270 |  |  |  |
| **Intraclass correlation** |  |  |  |  |  |  |  |  |  |  |
| Household | 0.185 | 0.048 |  | 0.108 | 0.299 | 0.112 | 0.034 |  | 0.061 | 0.197 |
| Village | 0.021 | 0.012 |  | 0.007 | 0.063 | 0.031 | 0.013 |  | 0.014 | 0.068 |
| Obs. | 1832 |  |  |  |  | 1832 |  |  |  |  |
| Crude Global R^2^ | 0.402 |  |  |  |  | 0.331 |  |  |  |  |
| Marginal R^2^ | 0.050 |  |  |  |  | 0.193 |  |  |  |  |
| Conditional R^2^ | 0.226 |  |  |  |  | 0.292 |  |  |  |  |

^a^Estimate represents coefficients for fixed components and variances for random components.

Panel A presents a General Linear Latent and Mixed Model with Binomial family. Panel B presents a General Linear Latent and Mixed Model with Gaussian family. For Panels A and B, respectively, the variance components (empty) models are presented in Table S6 and S7. Coinfection indicated at least 1 EPG (eggs per gram) of both S. mansoni and hookworm with base (excluded category) or no infection or single S. mansoni and hookworm infections.

## Table J: Any *S. mansoni* and hookworm infections on dependent variable of anaemia

|  | **Odds ratio^a^** | **Clustered Std. Err.** | **p-value** | **95% CI** | |
| --- | --- | --- | --- | --- | --- |
| **Variable** |  |  |  |  |  |
| *S. mansoni* 1-99 EPG | 1.023 | 0.128 | 0.856 | 0.800 | 1.308 |
| *S. mansoni* 100-399 EPG | 1.413 | 0.256 | 0.056 | 0.991 | 2.015 |
| *S. mansoni* 400+ EPG | 2.433 | 0.471 | <0.001 | 1.666 | 3.555 |
| Hookworm 1-99 EPG | 1.197 | 0.158 | 0.174 | 0.924 | 1.551 |
| Hookworm 100-399 EPG | 0.890 | 0.146 | 0.478 | 0.645 | 1.228 |
| Hookworm 400+ EPG | 1.644 | 0.277 | 0.003 | 1.181 | 2.287 |
| Age | 0.983 | 0.003 | <0.001 | 0.977 | 0.989 |
| Female | 1.124 | 0.117 | 0.260 | 0.917 | 1.377 |
| Malaria in past 6 months | 0.749 | 0.075 | 0.004 | 0.615 | 0.911 |
| Business (owns small shop) | 0.654 | 0.144 | 0.053 | 0.425 | 1.006 |
| Fishing (fishermen and fishmongers) | 0.738 | 0.137 | 0.101 | 0.513 | 1.061 |
| Rice farmer | 0.796 | 0.130 | 0.163 | 0.578 | 1.096 |
| School teacher | 0.474 | 0.223 | 0.112 | 0.188 | 1.192 |
| Other employment | 1.027 | 0.228 | 0.905 | 0.664 | 1.587 |
| Lake site only within village (no beach, but small boat landing site on Lake Victoria) | 0.797 | 0.088 | 0.041 | 0.642 | 0.990 |
| 3 or more roads within village | 0.808 | 0.087 | 0.046 | 0.654 | 0.996 |
| Constant | 1.394 | 0.199 | 0.020 | 1.054 | 1.844 |
| Obs. 1832 |  |  |  |  |  |
| ^a^Multiple logistic regression with standard errors adjusted for 916 household clusters. Occupations are represented at the household-level and indicate the employment of the household head. | | | | | |

This model is specified with the same variables used for the main in-text anaemia model, but estimated in a simple multivariate logistic regression. The estimates from this model were used to calculate adjusted population attributable fractions for elimination of heavy *S. mansoni* or hookworm infection.

## Table K: Heavy *S. mansoni* and hookworm infections on dependent variable of anaemia

|  | **Odds ratio^a^** | **Clustered Std. Err.** | **p-value** | **95% CI** | |
| --- | --- | --- | --- | --- | --- |
| **Variable** |  |  |  |  |  |
| *S. mansoni* 400+ EPG | 2.311 | 0.429 | <0.001 | 1.606 | 3.326 |
| Hookworm 400+ EPG | 1.612 | 0.261 | 0.003 | 1.173 | 2.214 |
| Age | 0.982 | 0.003 | <0.001 | 0.976 | 0.988 |
| Female | 1.114 | 0.115 | 0.298 | 0.909 | 1.364 |
| Malaria in past 6 months | 0.753 | 0.075 | 0.004 | 0.619 | 0.915 |
| Business (owns small shop) | 0.646 | 0.141 | 0.045 | 0.421 | 0.991 |
| Fishing (fishermen and fishmongers) | 0.766 | 0.142 | 0.152 | 0.532 | 1.103 |
| Rice farmer | 0.803 | 0.131 | 0.179 | 0.584 | 1.105 |
| School teacher | 0.462 | 0.206 | 0.083 | 0.193 | 1.106 |
| Other employment | 1.019 | 0.227 | 0.933 | 0.659 | 1.576 |
| Lake site only within village (no beach, but small boat landing site on Lake Victoria) | 0.791 | 0.087 | 0.033 | 0.638 | 0.981 |
| 3 or more roads within village | 0.794 | 0.085 | 0.031 | 0.644 | 0.979 |
| Constant | 1.520 | 0.200 | 0.001 | 1.175 | 1.967 |
| Obs. 1832 |  |  |  |  |  |
| **^a^** Multiple logistic regression with standard errors adjusted for 916 household clusters. Occupations are represented at the household-level and indicate the employment of the household head. | | | | | |

The estimates from this model were used to calculate adjusted population attributable fractions for reducing intensity of *S. mansoni* or hookworm infections. The potential impacts of the reduction of heavy S. mansoni to uninfected/low/moderate S. mansoni and the reduction of heavy hookworm to uninfected/low/moderate hookworm were measured.

## References

1. Menard S. Applied logistic regression analysis: Sage; 2002.

2. O'brien R. A Caution Regarding Rules of Thumb for Variance Inflation Factors. Qual. Quant. 2007;41:673-90.
